# Race Correction and Algorithmic Bias in Atrial Fibrillation Wearable Technologies

**DOI:** 10.1089/heq.2023.0034

**Published:** 2023-11-30

**Authors:** Beza Merid, Vanessa Volpe

**Affiliations:** ^1^ School for the Future of Innovation in Society, Arizona State University, Tempe, Arizona, USA.; ^2^ Department of Psychology, North Carolina State University, Raleigh, NC.

**Keywords:** African American, cardiovascular health, health disparities, technology

## Abstract

Stakeholders in biomedicine are evaluating how race corrections in clinical algorithms inequitably allocate health care resources on the basis of a misunderstanding of race-as-genetic difference. Ostensibly used to intervene on persistent disparities in health outcomes across different racial groups, these troubling corrections in risk assessments embed essentialist ideas of race as a biological reality, rather than a social and political construct that reproduces a racial hierarchy, into practice guidelines. This article explores the harms of such race corrections by considering how the technologies we use to account for disparities in health outcomes can actually innovate and amplify these harms. Focusing on the design of wearable digital health technologies that use photoplethysmographic sensors to detect atrial fibrillation, we argue that these devices, which are notoriously poor in accurately functioning on users with darker skin tones, embed a subtle form of race correction that presupposes the need for explicit adjustments in the clinical interpretation of their data outputs. We point to research on responsible innovation in health, and its commitment to being responsive in addressing inequities and harms, as a way forward for those invested in the elimination of race correction.

## Introduction

In the world of digital health, promises that innovative technologies will fundamentally transform the delivery of health care abound. The arguments for the promise of these technologies often include the increased efficiency of new digital tools, the presumed objectivity of automated forms of risk assessment, and the opportunity to further efforts to eliminate racial health disparities and move closer toward health equity. This article provides a case study of one such category of digital health technologies instantiating this promise: consumer wearable devices intended to help users detect a cardiovascular concern called atrial fibrillation (AF), one type of irregular heart rhythm.

Wearable monitors in this category are part of a larger push within cardiovascular care, among other fields of medicine, relying on technologies deploying algorithmic prediction to help users assess prediagnostic disease risk outside of clinical settings, participate in medical decision making, and, as a result, address barriers in access to care as a driver of disparities in health outcomes. Although these technologies may improve users' capacity to begin assessing disease risk at home and to accumulate data that can inform decision making about intervention strategies, these technologies are far more limited in their capacity to address the persistence of inequities in disease incidence and health outcomes.

Driving this limitation, ironically, are the means by which these devices and biomedicine situate the consideration of race in assessing disease risk. Here we focus on wearable monitors that rely on photoplethysmographic (PPG) sensors, a technology that measures light absorption against the skin. PPG sensors are beset by algorithmic bias, and are notoriously poor in accurately functioning on users with darker skin tones. Many PPG sensors rely on green light signaling as a way to detect biological signals below the skin to index bodily functioning (e.g., heart rate, blood pressure, and oxygen saturation).

However, they demonstrate technical algorithmic bias in that green light signaling cannot accurately detect these biological signals for those who do not have lighter skin tones.^1^ Despite this algorithmic bias, PPG sensors are embedded in wearable devices that are regularly marketed to and used by individuals with darker skin tones (e.g., Fitbit, see Case Study: Wearable Devices for AF Detection below). This article looks to advance the current understanding of algorithmic bias in these wearable devices by situating these inaccuracies in the operation of PPG sensors across a variety of racial and ethnic groups as a subtle unacknowledged form of race correction already embedded in these devices.

Drawing on an examination of how a misunderstanding of what race is shapes how biomedicine works to intervene on disparities in health outcomes, this article offers insights from the scholarly field of responsible innovation, which examines the human and social dimensions of innovation to provide guidance on how ethical, moral, sustainability, and equity challenges can be addressed in the development and deployment of technological innovation. To do so, we engage with research on responsible innovation in health (RIH), an evaluative framework developed in this field to assess the ethical and social dimensions of innovation in health care.

We draw on the RIH framework to articulate why health care providers and researchers should eliminate race correction in all forms, opting instead to shift the subject of inquiries about the causes of disparities in outcomes from race to racism. We also explore how these insights help us rethink our understanding of what technology can and cannot do to “solve” complex social problems, and how the allure of this kind of “technosolutionism” can prevent us from enacting necessary structural changes to how we deliver care.

## Race Correction, Clinical Algorithms, and Cardiovascular Care

The effort to close persistent racial disparities in health outcomes for cardiovascular care patients is a pressing one. Cardiovascular disease is a leading cause of death for all populations in the United States, and there are profound racial disparities in the incidence of disease and access to appropriate treatment and surgical procedures as a result of wide variability in both the quality of medical facilities and care rendered. Because of this variability, individuals from racially marginalized populations have higher rates of cardiovascular morbidity and mortality.^2,3^

These disparities result in delays in treatment and referrals for specialty care as well as missed diagnoses, which can have devastating effects on patients' overall health outcomes. Acknowledging the urgency of the need to intervene, one strategy deployed within biomedicine to address these kinds of disparities is the adoption of algorithms and practice guidelines that correct or adjust outputs of diagnostic technologies to presumably equitably distribute access to resources and interventions.

Known as “race correction,” this practice has a long and controversial history, complicating risk assessment and disease diagnosis. For example, in the use of spirometers, popularized in the 19th and early 20th centuries, this technology used to evaluate patients for abnormal lung functioning employs race corrections to account for differences in lung capacity between racialized population groups.

Built on a history of scientific racism in the 19th century that sought to explain poor lung functioning in Black patients as the result of a natural racial inferiority and codified in the 1970s to explain disparities in lung functioning between Black and White asbestos workers in the United States, the use of a blanket race correction factor for Black patients in spirometry essentializes differences in lung capacity that may reflect social and environmental factors by treating them as the product of innate biological differences.

These problems also exist in the use of estimated glomerular filtration rate (eGFR), which measures kidney functioning and informs the management of kidney transplantation. The use of race correction in eGFR is based on data from a small sample of Black patients who participated in chronic kidney disease studies from the 1970s and 1990s. In these small samples, Black participants were shown to have higher creatinine excretion rates than White participants; these findings prompted researchers to conclude that Black race was the driver of these higher rates and that a blanket correction factor was necessary to properly identify pathology in kidney function.^4–6^

Taking patients' self-reported race as determinative of their specific risk of disease, this practice enables providers to make automatic adjustments in calculating risk scores that are used to inform providers' clinical assessments of patients' physical capacity and decisions about their likelihood of surgical complications and in-hospital mortality. The problem with race correction is that these automatic adjustments based on race can erroneously prompt providers to regard patients as being at low risk for disease or complication. Once these patients are regarded as being at lower risk, they may have to reach a much higher threshold during risk assessment to access clinical resources and treatment.

When race corrections are applied to Black patients (i.e., individuals racialized as Black in the United States based on phenotypic characteristics who share a sociopolitical history of disenfranchisement and marginalization), appearing to be at lower risk of disease or complication can make it harder to access care and can worsen existing disparities in health outcomes in turn.^4,7^

Race correction is founded on an essentialist misunderstanding of race as a biological reality rather than a social construct. It situates disparities in health outcomes as the product of inherent biological differences between racialized populations. Within this view, racial groups comprise “discrete genetic categories” that are “biogenetically similar.”^8^

When these presumed genetic differences between populations are taken to be determinative of not only health outcomes but also social inequality, this essentialist view of race is then operationalized to develop mathematical rules, or corrections, that purport to adjust measurements of relative disease risk by population through the application of fixed and quantifiable numerical proxies for racial identity. These corrections ignore the sociopolitical complexity of race, the fluidity of racial identification, and the role that racism plays in structuring unequal access to and delivery of health care.^9^

One use for such corrections in risk scores is to account for disparities in the prevalence of diagnosis among a racial or ethnic group relative to the presence of risk factors for that disease. In the case of AF, for example, Black patients have a lower prevalence of diagnosis despite having more risk factors, including hypertension, diabetes mellitus, and heart failure, than White patients.^10–12^ Known as the “AF ethnic paradox,”^10^ this phenomenon has driven clinicians and researchers to search for “novel genetic loci”^12^ in people identified as Black that can explain this disparity, and has promoted the development of predictive risk scoring models that seek to analyze patients' race and ethnicity in search of a culprit.^10^ We are unaware of any efforts to address this paradox by examining implicit biases or discriminatory behaviors of diagnosing providers.

Despite the ostensibly well-meaning motivation behind this search for a biological cause for disparate health outcomes, race correction does away with the nuance required to understand and meaningfully act on inequities in health. Rather than serve to effectively correlate a patient factor such as race or ethnicity with the ultimate health outcome that patients experience, race correction assumes race to be a static category, thereby essentializing biological difference between populations.

This practice represents “a failure to understand the meaning of race and its connection to racism,” focusing on a superficial understanding of this fluid, “socially constructed grouping” rather than attending to the structural harms that reproduce racial health disparities.^4^ Indeed, race is a social category that has come to represent phenotypic differences between groups of people in our society. This categorization of people into “races” is sociopolitical, created to maintain a social hierarchy for the purpose of assigning differential access and resources to groups.^13,14^ Race correction replicates essentialist notions of race as biological by considering self-reported race as a construct removed from its sociopolitical and theoretical context.

Given this propensity to situate race as a disease risk factor without properly accounting for what race is and, more accurately, how racism shapes health outcomes, we are interested in what happens to Black patients at risk of developing cardiovascular concerns when these kinds of corrections are used to interpret the data wearable monitors produce about potential cardiovascular irregularities. In the next section, we use wearable devices that detect AF as a case study, and we examine how these wearable devices function and the harms that a failure to function properly among Black patients can introduce.

## Case Study: Wearable Devices for AF Detection

AF is an irregular heart rhythm, in which the atria beat out of sync with the ventricles, leading to decreased blood flow, blood clots, cardiovascular risk,^15^ and excess mortality rates at the population level.^16^ This irregular rhythm accounts for one quarter of strokes^17^ and can be asymptomatic or subclinical,^18^ making it especially dangerous and, therefore, important to identify in noninvasive ways before a cardiac event.^19^ Furthermore, although epidemiological research indicates that Black Americans have lower incidence and prevalence of AF,^20^ concerns about proportionate recruitment of Black Americans into AF clinical trials and clinical underdetection or diagnosis of AF suggest that the impact of AF for Black Americans may be underestimated.^2,21^

For these reasons, wearable devices that can detect irregular heart rhythms such as AF are a prime focus for development and deployment, as they hold great potential for preventing stroke and serious cardiovascular events while also capturing rhythms over time during a monitoring period to potentially ascertain AF burden.^22^

Traditionally, ambulatory wearable monitoring of electrocardiogram (ECG) waveforms has been limited to clinical practice and research, employed under the guidelines for rigorously tested, federally recognized, and approved medical devices. However, with a rise in direct-to-consumer health, wellness, and fitness wearable devices writ large (e.g., Apple Watch, FitBit, QardioCore, Mio Alpha, Polar watches), corporations and medical technologists have been increasingly interested in using these more affordable consumer wearables to detect AF.^23^

Consumer wearable devices capture and estimate a variety of cardiac signals, including heart rate, heart rhythm, and even thoracic parameters through sensors embedded in the devices.^24^ A majority of consumer wearables that advertise the ability to detect AF are wrist-worn devices that utilize PPG sensors.^25–27^ These sensors estimate heart rate and blood flow as a function of light absorption detected against the skin. In the case of wrist-worn bands, photodetectors capture changes in blood volume from peripheral pulses at the wrist.

Subsequent to that data capture, algorithms are used to process sensor data and assign them cardiovascular meaning. In the case of AF, the wrist-worn sensor estimates an ECG waveform from peripheral pulse. This waveform is then passed through a proprietary algorithm to identify portions of the ECG that evidence key signatures of the irregular heart rhythm of AF.

The promise of these devices and their corresponding AF detection algorithms is situated within the context of the pursuit of health equity, including but not limited to expansion of personal access to health information and lower cost than standard medical-grade ambulatory cardiac monitoring devices.^28^ These devices and algorithms are gaining momentum among clinicians since FDA approval for the use of AF detection algorithms for wearable devices was passed;^29^ a new FDA category of “Software as a Medical Device” (SaMD), which allows medical applications on nonmedical devices such as FitBits, was introduced;^30^ and the European Society for Cardiology mentioned opportunities for wearables to aid in AF diagnosis in 2020.^31^ Although not yet widely used in clinical practice, their use is also increasing with recent advances in telehealth in the wake of COVID-19, and they are largely seen as reputable options for arrythmia screening and diagnosis.^32^

They are also gaining momentum among patients in the direct-to-consumer marketplace. Approximately 120 million Americans had a FitBit in 2022^33^ and >2 million people are using FitBit's AF detection algorithm as of June 2022.^34^ Even though many users know their risk of AF is low, they continue to regularly screen by using such algorithms, making AF screening part of their everyday health practice.^35^ One lawsuit further demonstrates consumers' belief in the accuracy of such products as marketed. In a 2016 lawsuit against FitBit, the plaintiffs alleged that two FitBit devices undercounted users' heart rates during exercise, which created risk of overexertion.^36^ The plaintiffs argued that FitBit had made false claims about accuracy of the devices' heart rhythm detection and suppressed these data from studies of different activity levels to mislead consumers. In general, the AF detection algorithm relies on the same PPG technology.

Several interrelated problems exist with algorithmic detection of AF that are germane to concerns of health equity. First, the use of PPG means that these wearables and their concomitant AF algorithms operate poorly on darker skin tones. Second, industry decisions about the populations included within and excluded from benchmark data sets on which algorithms are trained and tested for accuracy and specificity pose a concern about exclusion and erasure of Black populations from cutting edge technological innovations, and may potentially make the use of such innovations inaccurate or dangerous for, Black users who have darker skin tones. Third, the proprietary nature of AF algorithms makes it challenging to conduct equity audits, and there are associated concerns about data privacy and security. Fourth, AF algorithms deployed by wearables may inadvertently exacerbate existing structural inequities in access to technology and health care. This all translates into increased likelihood of undetected AF disease risk and worse health outcomes systematically for Black populations.

The accuracy of existing AF algorithms has been established through clinical trials^37^ such as the Fitbit Heart Study (conducted 2020, results presented at the American Heart Association in 2021). This study provided clinical validation for the algorithm.^38,39^ AF detection accuracy was assessed by providing participants for whom the algorithm detected AF with a 1-week traditional ECG ambulatory monitor used in clinical care that captures an ECG waveform from electrodes placed on the skin, rather than approximations of pulse pressure and PPG technology. In total, 98.2% of participants' FitBits (through irregular rhythm notifications) and ECG patches detected AF at the same time. However, only 32% of those who received notification of an irregular heart rhythm had AF detected by the patch. This may not entirely be problematic, as it may be more clinically meaningful to be more conservative in flagging potential AF. However, others suggest that these devices increase the risk of medical overuse, as repeated screening among those with minimal disease risk increases clinical encounters that lead to overdiagnosis, overtreatment, and low value care.^35^

Importantly, although study authors confirmed that detection of AF results was relatively consistent across age groups, they did not investigate consistency across racial groups. This is concerning given that wearables utilizing PPG are less accurate for individuals with different skin tones, as PPG utilizes light absorption to index pulse waves.^40,41^ Most PPG devices use green light due to the high absorption spectrum of hemoglobin, yet, as we note earlier, the absorption of green light by melanin for those with darker skin limits light penetration and gives inaccurate readings.^40^

If we take seriously the established racial inequities in the accurate operation of PPG technology with non-White racial and ethnic groups with relatively darker skin tones, we can see how these inequities become instantiated and replicated such that AF detection is not as possible for Black populations. Given these inequities in accuracy, it is unclear that these wearable consumer devices improve access to health information for patients in racially marginalized populations with darker skin tones.

## Algorithmic Bias as Race Correction

The danger posed by this inaccuracy in algorithmic detection is in its normalization of both the erasure of marginalized populations, explored above, and the presupposition that a technological fix can remedy this harm. In this case, there are two “technologies” at play. The first, as we have discussed, is the system of adjustments in risk scoring that comprise race correction. The second technology is race itself.

Ongoing debates in biomedicine about whether and how to embed considerations of race into practice guidelines^4,7^ take race to be a static reflection of immutable differences between populations; in fact, race should be understood as “a social classification whose delineations change across time, geography, and political priorities.”^4^ So viewed, we can begin to understand race as a kind of technology that, as Benjamin^6^ writes, creates parallel social universes and premature death, functioning as a conduit for the innovation of inequity.

Race, Benjamin^42^ writes elsewhere, is a tool “designed to stratify and sanctify social injustice as part of the architecture of everyday life.” Its function in this context is to encode difference as essential, and as its own rationale for the “enforcement of racial hierarchies with real consequences.”^42^ Our concern here is that, within this parallel social universe, a reliance on “technology” and the use of essentialist race corrections could supplant more substantive engagements with racism as a driver of disease disparities.

Wearable technologies such as the AF detection monitors discussed here are rightly hailed for enabling patients to better understand and participate in health decision making. But when essentialist misunderstandings of what race is and how it shapes health are uncritically reproduced in the design of these tools, even our most well-intentioned interventions remain limited in their capacity to address structural barriers to health. Wearable devices do not exist outside of the social and political contexts that shape and reproduce the disparate health outcomes they purport to address; to act otherwise is to miss an opportunity to imagine more transformative, although challenging, interventions to pursue improved health and well-being for all people.

In the section that follows, we turn to the field of responsible innovation to explore how the values embedded within the design of innovative technologies can be better aligned with the needs of racially marginalized patient populations.

## Looking to the RIH Framework for Solutions

As we work to imagine alternative futures where the architecture of digital health technologies is no longer founded on the practice of race correction, we must also pay attention to the architecture of innovation in biomedicine itself. To this end, research from Silva et al.^43^ introducing a policy-oriented framework for RIH is particularly instructive. Building on a field of study known as responsible innovation, which establishes anticipation of and responsiveness to the harms of innovation as core principles of governance, RIH enables stakeholders to work collaboratively to abide by a set of ethical and social principles when designing, financing, producing, distributing, and using sociotechnical solutions that address the needs and challenges of a health care system.^43^

The RIH framework presents a series of questions for stakeholders to answer within five value domains: population health, health system, economic, organizational, and environmental. These questions prompt stakeholders to examine whether an innovation addresses a relevant health problem; whether it does so in a way that promotes health equity; and, in particular, whether an innovation is developed seeking to mitigate ethical, legal, and social issues related to its design and use.

We argue that the RIH framework should be integrated into the production and testing of wearable digital health technologies. By fostering a system through which innovations and practices that engender the kind of harm attributed to race correction can be deemed irresponsible and excluded from further development and use, we can continue the work of assessing the persistence of racial disparities in diagnosis and health outcomes without relying on reductionist conceptions of race as a driver of disease disparities. In centering this kind of responsiveness to algorithmic bias, as Silva et al. write, we can also maintain focus on the “value of providing flexible and opportune solutions to existing and emerging system-level challenges.”^43^

Race correction as a practice is already widespread, and its harms are and have been widely distributed. We argue that the adoption of an evaluative framework such as RIH can equip us to reckon with these harms, to establish regulatory mechanisms that recognize how this practice embeds racism in health care, and, critically, to create a means by which we end this practice and the use of technologies that perpetuate it.

Within the field of science and technology studies, Stilgoe et al.^44^ write, it is acknowledged that “conceptions of responsibility should build on the understanding that science and technology are not only technically but also socially and politically constituted.” This sense of responsibility, and responsiveness to the social and political conditions through which both technologies and health inequities are produced, requires us to reckon with the fact that these tools are not ideologically neutral; they are not free of politics, untainted by the biases that so often limit our own capacity to sufficiently deliver care.

When race is embedded into the architecture of technologies such as the wearable devices we discuss, it is racism—rather than equity—that is being innovated. By working to align the processes of innovation that drive the development of these tools with societal values around health equity, we can begin to do the hard work of reconciling the goals of the health care providers and health services researchers who advance these innovations with the demands of our health care systems and the needs of vulnerable patient communities among whom these harms are inequitably burdensome.

## Conclusion

As we consider the harms of race correction within biomedicine writ large and the world of digital health in particular, we argue that these efforts should include attention to both the adjustments we make in interpreting risk assessment data and the algorithmic biases that shape these data outputs. In continuing to rely on reductionist understandings of race as a biological category to explain disparities in health outcomes, we miss an opportunity to look for structural interventions that address the persistent reproduction of these disparities in favor of adopting technological fixes for the data that reveal them.

By exploring how race functions as a technology that amplifies inequity and focusing on racism as a driver of disparities in health outcomes, we enable ourselves to address these harms with far greater nuance.

Adopting an interventionist framework such as RIH that can recognize the importance of responsiveness to algorithmic bias as a subtle form of race correction gives us the tools we need to regulate these innovative technologies, and to develop a future-oriented ethic of care that centers values such as health equity in the design of these devices, working to prevent these harms rather than simply account for them after the fact.

## Abbreviations Used

AF: atrial fibrillation
ECG: electrocardiogram
eGFR: estimated glomerular filtration rate
PPG: photoplethysmographic
RIH: responsible innovation in health

## References

[1] Astorino S, Simmonds M, Puget J-F, et al. Artificial Intelligence: Evolution and Revolution. MC Press: Bose, ID; 2019.

[2] Capers Q IV, Sharalaya Z. Racial disparities in cardiovascular care: A review of culprits and potential solutions. J Racial Ethn Health Disparities 2014;1:171–180.

[3] Heckbert SR, Austin TA, Jensen PN, et al. Differences by race/ethnicity in the prevalence of clinically detected and monitor-detected atrial fibrillation. Circ Arrhythm Electrophysiol 2020;13:24–28. 10.1161/CIRCEP.119.007698 PMC7204495 31934795

[4] Roberts D. Abolish race correction. Lancet 2021;397:17–18. 33388099 10.1016/S0140-6736(20)32716-1

[5] Braun L. Breathing Race into the Machine: The Surprising Career of the Spirometer from Plantation to Genetics. University of Minnesota Press: Minneapolis, MN; 2014.

[6] Benjamin R. Innovating inequity: If race is a technology, postracialism is the genius bar. Ethn Racial Stud 2016;39(13): 2227–2234.

[7] Vyas DA, Eisenstein LG, Jones DS. Hidden in plain sight: Reconsidering the use of race correction in clinical algorithms. N Engl J Med 2020;383(9):874–882. 32853499 10.1056/NEJMms2004740

[8] Tsai J. How should educators and publishers eliminate racial essentialism? AMA J Ethics 2022;24(3):E201–E211. 35325521 10.1001/amajethics.2022.201

[9] Richmond II SP, Grubbs V. How abolition of race-based medicine is necessary to American health justice. AMA J Ethics 2022;24(3):E226–E232. 35325524 10.1001/amajethics.2022.226

[10] Kowlgi GN, Gunda S, Padala SK, et al. Comparison of frequency of atrial fibrillation in Blacks versus Whites and the utilization of race in a Novel Risk Score. Am K Cardiol 2020;135:68–76. 10.1016/j.amjcard.2020.08.029 32866451

[11] Nanda A, Kabra R. Racial differences in atrial fibrillation epidemiology, management, and outcomes. Curr Treat Options Cardiovasc Med 2019;21(12):85. 31820122 10.1007/s11936-019-0793-5

[12] Stamos TD, Darbar D. The ‘Double’ paradox of atrial fibrillation in Black individuals. JAMA Cardiol 2016;1(4):377–379. 27438310 10.1001/jamacardio.2016.1259

[13] Bonilla-Silva E. Race without Racists (4th Ed.). Rowman & Littlefield Publishers: Seabrook, MD; 2013.

[14] Roberts DE. Fatal Invention: How Science, Politics, and Big Business Re-Create Race in the Twenty-First Century. The New Press: New York, NY; 2012.

[15] Calkins H, Brugada J, Packer DL, et al. HRS/EHRA/ECAS Expert Consensus Statement on catheter and surgical ablation of atrial fibrillation: Recommendations for Personnel, Policy, Procedures and Follow-up. Heart Rhythm 2007;4:1–46. 17556213 10.1016/j.hrthm.2007.04.005

[16] Benjamin EJ, Wolf PA, D'Agostino RB, et al. Impact of atrial fibrillation on the risk of death: The Framingham Heart Study. Circulation 1998;98:946–952. 9737513 10.1161/01.cir.98.10.946

[17] Marini C, De Santis F, Sacco S, et al. Contribution of atrial fibrillation to incidence and outcome of ischemic stroke: Results from a population-based study. Stroke 2005;36(6):1115–1119. 15879330 10.1161/01.STR.0000166053.83476.4a

[18] Lin HJ, Wolf PA, Benjamin EJ, et al. Newly diagnosed atrial fibrillation and acute stroke: The Framingham Study. Stroke 1995;26(9):1527–1530. 7660392 10.1161/01.str.26.9.1527

[19] Zakeri R, Wagoner DRV, Calkins H, et al. The burden of proof: The current state of atrial fibrillation prevention and treatment trials. Heart Rhythm 2017;14(5):763–782. 28161513 10.1016/j.hrthm.2017.01.032 PMC5403606

[20] CDC. Atrial Fibrillation Fact Sheet. Available from: https://www.cdc.gov/heartdisease/atrial_fibrillation.htm; https://medbox.iiab.me/modules/en-cdc/www.cdc.gov/dhdsp/data_statistics/fact_sheets/fs_atrial_fibrillation.htm [Last accessed: October 14, 2022].

[21] Amponsah MKD, Benjamin EJ, Magnani JW. Atrial fibrillation and race—A contemporary review. Curr Cardiovasc Risk Rep 2013;7(5):336–345; doi: 10.1007/s12170-013-0327-8 PMC3815541 24198867

[22] Chen LY, Chung MK, Allen LA, et al. Atrial fibrillation burden: Moving beyond atrial fibrillation as a binary entity: A Scientific Statement from the American Heart Association. Circulation 2018;137:e623–e644. 29661944 10.1161/CIR.0000000000000568 PMC8463258

[23] Raja JM, Elsakr C, Roman S, et al. Apple watch, wearables, and heart rhythm: Where do we stand? Ann Trans Med 2019;7(17):417. 10.21037/atm.2019.06.79 PMC6787392 31660316

[24] Pevnick JM, Birkeland K, Zimmer R, et al. Wearable technology for cardiology: An update and framework for the future. Trends Cardiovasc Med 2018;28(2):144–150. 28818431 10.1016/j.tcm.2017.08.003 PMC5762264

[25] Cheung CC, Krahn AD, Andrade JG. The emerging role of wearable technologies in detection of arrhythmia. Can J Cardiol 2018;34(8):1083–1087. 30049358 10.1016/j.cjca.2018.05.003

[26] Ip JE. Evaluation of cardiac rhythm abnormalities from wearable devices. JAMA 2019;321(11):1098–1099. 30874744 10.1001/jama.2019.1681

[27] Ip JE. Wearable devices for cardiac rhythm diagnosis and management. JAMA 2019;321(4):337–338. 30633301 10.1001/jama.2018.20437

[28] Ioannidis DC, Kapasouri EM, Vassiliou VS. Wearable devices: Monitoring the future? Oxf Med Case Rep 2019;12:492–494. 10.1093/omcr/omz143 PMC6937450 31908818

[29] US Food and Drug Administration. De Novo Classification Request for Irregular Heart Rhythm Notification Feature (DEN180042, Apple Inc.). August 8, 2018; 2018. Available from: https://www.accessdata.fda.gov/cdrh_docs/reviews/DEN180042.pdf [Last accessed: April 26, 2022].

[30] U.S. Food and Drug Administration, 2019. Device Software Functions Including Mobile Medical Applications. Available from: https://www.fda.gov/medical-devices/digital-health-center-excellence/device-software-functions-including-mobile-medical-applications [Last accessed: September 7, 2023].

[31] Hindricks G, Potpara T, Dagres N, et al. 2020 ESC Guidelines for the diagnosis and management of atrial fibrillation developed in collaboration with the European Association for Cardio-Thoracic Surgery (EACTS): The Task Force for the diagnosis and management of atrial fibrillation of the European Society of Cardiology (ESC) Developed with the special contribution of the European Heart Rhythm Association (EHRA) of the ESC. Eur Heart J 2021;42(5):373–498. 32860505 10.1093/eurheartj/ehaa612

[32] Bayoumy K, Gaber M, Elshafeey A, et al. Smart wearable devices in cardiovascular care: Where we are and how to move forward. Nat Rev Cardiol 2021;18:581–599. 33664502 10.1038/s41569-021-00522-7 PMC7931503

[33] Statistica. Number of registered users of Fitbit from 2014 to 2021; 2022. Available from: https://www.statista.com/statistics/1327371/fitbit-registered-users/ [Last accessed: October 30, 2023].

[34] Schoon B. (9to5google). Fitbit Has Over 2 Million Users Already Enrolled in Its AFib Detection Feature; June 16, 2022. Available from: https://9to5google.com/2022/06/16/fitbit-afib-users [Last accessed: October 30, 2023].

[35] Shih P, Prokopovich K, Degeling C, et al. Direct-to-consumer detection of atrial fibrillation in smartwatch electrocardiogram: Medical overuse, medicalization and the experience of consumers. Soc Sci Med 2022;303:114954. 35569232 10.1016/j.socscimed.2022.114954

[36] McLellan v Fitbit. 3 16-cv-0036-JD (United States District Court Northern District of California, San Francisco); 2018.

[37] Cadmus-Bertram L, Gangnon R, Wirkus EJ, et al. The accuracy of heart rate monitoring by some wrist-worn activity trackers. Ann Intern Med 2017;166(8):610–612. 10.7326/L16-0353 PMC5564399 28395305

[38] Lubitz SA, Faranesh AZ, Atlas SJ, et al. Rationale and design of a large population study to validate software for the Assessment of Atrial Fibrillation from Data Acquired by a Consumer Tracker or Smartwatch: The Fitbit Heart Study. Am Heart J 2021;238:16–26. 33865810 10.1016/j.ahj.2021.04.003

[39] Lubitz SA, Faranesh AZ, Selvaggi C, et al. Detection of atrial fibrillation in a large population using wearable devices: The Fitbit Heart Study. Circulation 2022;146(19):1415–1424. 36148649 10.1161/CIRCULATIONAHA.122.060291 PMC9640290

[40] Fine J, Branan KL, Rodriguez AJ, et al. Sources of inaccuracy in photoplethysmography for continuous cardiovascular monitoring. Biosensors 2021;11(4):11040126. 10.3390/bios11040126 PMC8073123 33923469

[41] Nowara EM, McDuff D, Veeraraghavan A. A Meta-analysis of the Impact of Skin Tone and Gender on Non-Contact Photoplethysmography Measurements. Proceedings of the IEEE/CVF Conference on Computer Vision and Pattern Recognition (CVPR) Workshops, Seattle, WA; 2020; pp. 284–285.

[42] Benjamin R. Race After Technology: Abolitionist Tools for the New Jim Code. Polity Press: Medford, MA; 2019.

[43] Silva HP, Lehoux P Miller, et al. Introducing responsible innovation in health: A policy-oriented framework. Health Res Policy Syst 2018;16(90):1–13. 30200985 10.1186/s12961-018-0362-5 PMC6131953

[44] Stilgoe J, Owen R, Macnaghten P. Developing a framework for responsible innovation. Res Policy 2013;42:1568–1580.

